# Lindane Bioremediation Capability of Bacteria Associated with the Demosponge *Hymeniacidon perlevis*

**DOI:** 10.3390/md15040108

**Published:** 2017-04-06

**Authors:** Stabili Loredana, Pizzolante Graziano, Morgante Antonio, Nonnis Marzano Carlotta, Longo Caterina, Aresta Antonella Maria, Zambonin Carlo, Corriero Giuseppe, Alifano Pietro

**Affiliations:** 1 Istituto per l’Ambiente Marino Costiero, Unità Operativa di Supporto di Taranto, CNR, Via Roma 3, 74123 Taranto, Italy; 2 Dipartimento di Scienze e Tecnologie Biologiche ed Ambientali, Università del Salento, Via Prov.le Lecce Monteroni, 73100 Lecce, Italy; graziano.pizolante@unisalento.it (P.G.); anto.mrg88@gmail.com (M.A.); pietro.alifano@unisalento.it (A.P.); 3 Dipartimento di Biologia, Università di Bari Aldo Moro, 70125 Bari, Italy; carlotta.nonnismarzano@uniba.it (N.M.C.); caterina.longo@uniba.it (L.C.); giuseppe.corriero@uniba.it (C.G.); 4 CoNISMa, Piazzale Flaminio 9, 00196 Roma, Italy; 5 Dipartimento di Chimica, Università di Bari Aldo Moro, 70125 Bari, Italy; antonellamaria.aresta@uniba.it (A.A.M.); carlo.zambonin@uniba.it (Z.C.)

**Keywords:** *H. perlevis*, sponge-associated bacteria, gas chromatography–mass spectrometry (GC–MS), lindane, 1, 3–6-pentachloro-cyclohexene, bioremediation

## Abstract

Lindane is an organochlorine pesticide belonging to persistent organic pollutants (POPs) that has been widely used to treat agricultural pests. It is of particular concern because of its toxicity, persistence and tendency to bioaccumulate in terrestrial and aquatic ecosystems. In this context, we assessed the role of bacteria associated with the sponge *Hymeniacidon perlevis* in lindane degradation. Seven bacteria isolates were characterized and identified. These isolates showed a remarkable capacity to utilize lindane as a sole carbon source leading to a percentage of residual lindane ranging from 3% to 13% after 12 days of incubation with the pesticide. The lindane metabolite, 1,3–6-pentachloro-cyclohexene, was identified as result of lindane degradation and determined by gas chromatography–mass spectrometry (GC–MS). The bacteria capable of lindane degradation were identified on the basis of the phenotypic characterization by morphological, biochemical and cultural tests, completed with 16S rDNA sequence analysis, and assigned to *Mameliella phaeodactyli*, *Pseudovibrio* *ascidiaceicola*, *Oceanicaulis stylophorae*, *Ruegeria atlantica* and to three new uncharacterized species. The results obtained are a prelude to the development of future strategies for the in situ bioremediation of lindane.

## 1. Introduction

Over the last few decades, the important growth of industrialization, the intensive use of soils for agriculture and the continuous urban development [1] have released either directly or indirectly in the marine environment several kinds of pollutants without the adequate treatment to remove their harmful effects, thus determining the occurrence of serious pollution problems [2,3]. Nowadays, compounds of anthropogenic origin foreign to certain biological systems, namely xenobiotics, are found even more frequently with the majority of them showing harmful behaviour against the biota of these biological systems [4,5]. Among these compounds persistent organic pollutants (POPs) are included. POPs, as defined by the Governing Council of the United Nations Environment Program (UNEP) are “chemical substances that persist in the environment, bio-accumulate to hazardous levels in living organisms through the food, and pose a risk of causing adverse effects to human health and the environment.”

Among POPs are included many pesticides that are employed to control the harmful effects of pests on agriculture productions. Lindane (γ—hexachlorocyclohexane or γ-HCH), for example, has long been one of the most widely used insecticides, becoming almost ubiquitous nowadays [6], resulting in a consistent number of contaminated waters and a significant impact on natural ecosystems [7,8]. Around 1945 the commercial production of lindane started, and between 1950 and 2000 it has been estimated that about 6 million tons have been produced globally with the maximum annual usage rising to about 300,000 tons in 1981 [9]. Lindane can enter the coast, marine and oceanic environments by a number of processes, however, the air-water gas exchange as well as run-off and leaching from soil seem to be the primary ways of deposition and accumulation in water bodies. Thus, the application of this pesticide has resulted in marine contamination of global dimensions [9,10,11]. This pesticide in aquatic systems tends to become associated with particulate matter and accumulates in bed sediments, which is highly toxic to aquatic organisms and eventually responsible for the reduction of the abundance and diversity of species. It causes increased mortality in fishes and benthic macro-invertebrates species, and results in changes in the zooplankton community structure [12,13]. The contamination of seawater can affect early embryonic development in sea organisms such as starfish [14], zebrafish [15] and sea urchins [16]. Lindane is also a carcinogenic endocrine disrupter and is known to exert damaging effects on the reproductive and nervous systems in mammals [17,18]. Due to the deleterious effect of lindane on the environment and human health, in 2009 its use was banned in 50 countries and restricted in 33 more under the Stockholm convention on Persistent Organic Pollutants [19]. Various bilateral and multilateral international agreements and treaties have addressed this pesticide, including, the Rotterdam Convention, the OSPAR Commission for the Protection of the Marine Environment of the Northeast Atlantic and the Stockholm Convention [11]. However, organochlorines, including lindane, are still used or stocked without control in developing countries [20,21]. In this framework, decontamination of lindane-polluted marine environments is needed and represents a very complex task. This is due to the recalcitrant nature of organochloride pesticides; they are strongly resistant to physical, chemical and biological degradation [22,23]. In this scenario, the Water Framework Directive (WFD by the Commission of the European Union: EU 2000/60/EC) has shifted its emphasis away from primarily monitoring chemicals to an approach that incorporates both chemical and ecological objectives and is designed to protect the structure and functions of aquatic ecosystems [24]. Scientists all over the world are working towards the development of remediation technologies including physical, chemical and biological remediation. Bioremediation is a potential technique for the biological treatment of industrial waste and contaminated environments [25,26]. Recent research has shown that, apart from the utilization of microorganisms for the biodegradation of target pollutants, aquatic ecosystems are home to several invertebrate species who deserve the definition of zooremediators [27] due to their ability to hyperaccumulate, stabilize or degrade pollutants. Among them, marine sponges have demonstrated the capability to remediate aquatic microbial pollution [28,29,30] and accumulate metals [31,32]. In addition, sponges host massive consortia of microorganisms within the mesohyl matrix that can amount up to 60% of their total biomass [33], exceeding that of seawater by two to three orders of magnitude [34], and significantly contributing to the host metabolism [35]. Several studies have examined the diversity of sponge-associated microbial communities by using cultivation-based approaches, and revealed that the microbial communities can be quite different among different species of sponges including mostly the phyla Actinobacteria, Bacteroidetes, Cyanobacteria, Firmicutes, Planctomycetes, Proteobacteria and Verrucomicrobia [36,37,38,39,40]. Furthermore, the advancement of molecular techniques such as 16S rRNA gene-based community analysis and fluorescent in situ hybridization (FISH) has increased our knowledge of the diversity of microorganisms associated with marine sponges [41]. Because sponge-bacteria interactions are widely distributed and, in some cases, specific to the host, it is generally believed that symbiotic interactions exist between sponges and microorganisms [42]. Symbiotic functions that have been attributed to microbial symbionts include nutrient acquisition, stabilization of the sponge skeleton, secondary metabolite production, and processing of metabolic waste and xenobiotics [42,43]. In this framework, a previous study by Aresta et al. [44] investigated the ability of the demosponge *Hymeniacidon perlevis* to bioremediate lindane-polluted seawater during in vitro experimentation. Sponges showed low mortality when exposed to a lindane concentration of 1 μg/L and were able to remove about 50% of the lindane content from seawater within 48 h. Furthermore, the authors assessed the role exerted in lindane degradation by bacteria isolated from the sponge. In the present work, we increased the knowledge on the *H. perlevis*-associated bacteria able to degrade lindane. In particular, those bacteria showing a remarkable remediating capacity were isolated and characterized, complementing culture-based with molecular methods, with the goal of inferring some environmental implications as well as developing a promising potential tool for low-cost in situ bioremediation (detoxification) of lindane-polluted marine environments.

## 2. Results

### 2.1. Screening of Lindane Degradation by Bacterial Isolates

**Figure 1:**
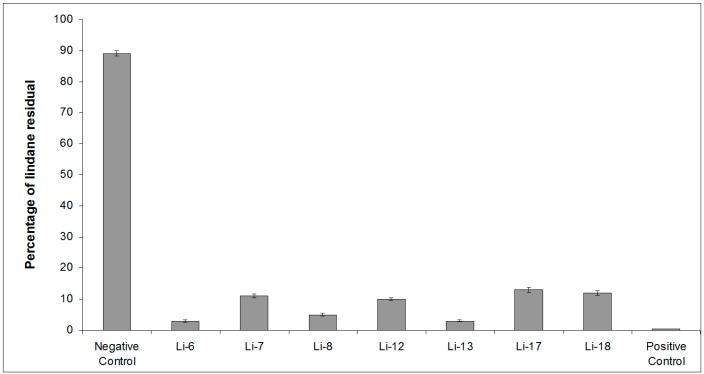
Percentage of residual lindane measured in all the tubes containing each bacterial strain incubated with lindane. Positive control = *Sphingobium japonicum* MTCC 6362 incubated with lindane. Negative control = lindane solution (0.05 mg/L).

**Figure 2:**
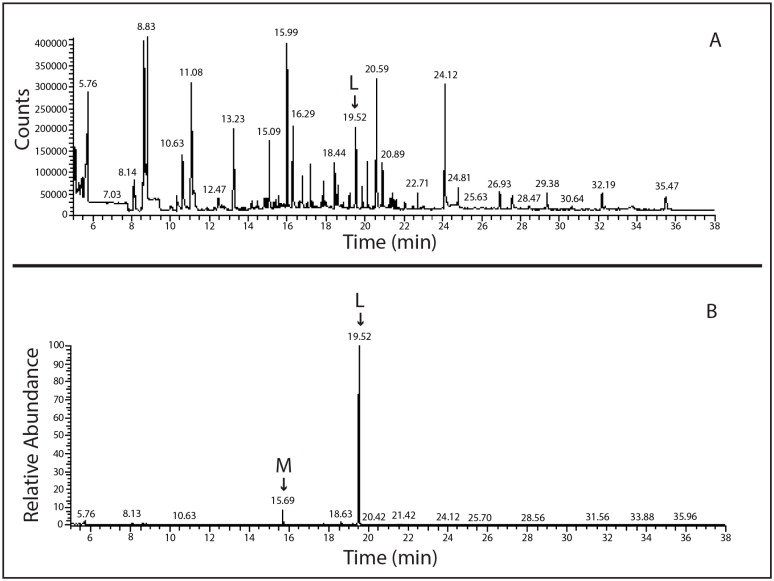
(**A**) Full scan SPME-GC-MS chromatogram of Li-13 sample after 12 days of incubation and (**B**) extracted-ion chromatogram at *m*/*z* 181 and 183; L: lindane peak (19.52 min); M: 1,3–6-pentachloro-cyclohexene (15.69 min).

In order to further characterize the lindane-degrading bacterial isolates arising from the sponge homogenates, we evaluated their ability to grow by utilizing lindane as a sole carbon source for 12 days. For this purpose, tubes containing lindane (0.05 mg/L) were inoculated with each bacterial isolate (named as Li-6, Li-7, Li-8, Li-12, Li-13, Li-17 and Li-18) and, after 12 days, were subjected to SPME-GC-MS analysis to evaluate the residual lindane concentration. *Sphingobium japonicum* MTCC 6362 incubated with lindane (0.05 mg/L) was used as a positive control, and tubes containing only the lindane solution in AS (seawater filtered on 0.22 µm pore size) at the same concentration represented the negative control. As reported in Figure 1, remarkable lindane degradation was evidenced. Indeed, the residual lindane percentage ranged between 13% for Li-17 and 3% for Li-6, while the lindane concentration in the negative control remained high and was around 0.4% in the positive control. All samples were then subjected to further SPME-GC-MS analysis in TIC mode, to search for the presence of metabolites of lindane. Moreover, the extracted-ion SPME-GC-MS chromatogram (*m*/*z* 181 and 183) obtained by analyzing the content of all the bacterial strains incubated with lindane showed another peak (15.69 min) besides that of lindane. The spectrum was recognized by the National Institute of Standards and Technology (NIST) library as belonging to 1,3–6-pentachloro-cyclohexene (PCCH) (Figure 2), a well-known lindane metabolite [45,46,47]. The probability of the match exceeded 86%.

### 2.2. Molecular Analysis of Lindane-Degrading Bacteria Isolated from Hymeniacidon perlevis

The amplified sequences coding for 16S rRNA of the lindane-degrading bacteria Li-6, Li-7, Li-8, Li-12, Li-13, Li-17 and Li-18 were compared with the closest sequences present in the Ezbio cloud server [48]. Phylogenetic relationships between the 16S rRNA gene sequences of our isolates and those of their closely related reference strains are shown in Figures S1 and S2 (of Electronic Supplementary Material). Comparison analysis of the 16S rRNA gene sequence of isolate Li-6 indicated that the closest relative of this strain was Alteromonas australica CIP 109921T [49] (Table 1). Due to its low similarity with this closest reference strain, the isolate Li-6 in the Neighbor-Joining (NJ) tree occupied a distinct phylogenetic position (Figure S1A).

The 16S rRNA gene sequence of Li-7 exhibited high identity (Table 1) with that of type strain *Mameliella phaeodactyli* KCTC 42178T [50]. This result was consistent with the taxonomic position of our isolate in the NJ tree (Figure S1B). 

Li-8 revealed an identity of 100% (Table 1) to *Pseudovibrio ascidiaceicola* NBRC 100514T [51], also confirmed by phylogenetic analysis (Figure S1C).

Li-12 appeared to be closely related (Table 1) to *Oceanicaulis stylophorae* LMG 2723T [52], and was clustered with the abovementioned reference strain in the NJ tree (Figure S1D).

Li-13 revealed an identity (Table 1) with the reference strain *Sulfitobacter dubius* KMM 3584T [53], and branched in a phylogenetic cluster comprising *S. dubius*, *Oceanibulbus indolifex* and *Sulfitobacter delicatus* (Figure S2A).

Li-17 exhibited 98.67% of homology (Table 1) with that of the type strain *Bacillus aquimaris* JCM 11544T [54], and clustered with *B. aquimaris* and *Bacillus vietnamensis* in the NJ tree (Figure S2B).

Lastly, phylogenetic analysis indicated that the closest reference strain of isolate Li-18 was *Ruegeria atlantica* NBRC 15792T [55] (Table 1). In the NJ tree, Li-18 was positioned together with the aforesaid reference strain (Figure S2C).

## 3. Discussion

In a previous study [44], we isolated some lindane-degrading bacteria associated with the demosponge *Hymeniacidon perlevis*. Due to their filtering habitus, sponges potentially accumulate contaminants from the environment [56,57]. Moreover, being sessile marine invertebrates and modular in body organization, they can live many years in the same location and therefore have the capability to accumulate anthropogenic pollutants over a long period. In particular, *H. perlevis* is a suitable species for this investigation, because it lives in environments subjected to strong anthropogenic pollution such as semi-enclosed basins and harbors, and is common and abundant along coastlines, thus providing abundant material to work with [30,58]. It is well known that almost all marine sponges host in their tissues a large number of microorganisms playing a central role in sponge biology [59]. Several observations support the idea that bacteria synthesize sponge-specific compounds either completely or in the form of precursors completed subsequently by sponge metabolism, and carry out several functions formerly ascribed to sponges [59]. In the present work, we further characterized and identified, for the first time, the lindane-degrading bacteria associated with the sponge *H. perlevis*. We demonstrated that the seven selected strains were able to grow well in presence of lindane as the only carbon source, leading to a lindane residual percentage that ranged from 13% to 3% after 12 days. It is well known that the aerobic degradation pathway of lindane is initiated with dechlorination to form PCCH, followed by further reactions leading to various products [47,60]. By using the SPME-GC-MS approach in the present study, we confirmed, as already reported by Aresta et al. [44], the presence of a small amount of PCCH, which was clear evidence of a bacteria-mediated degradation in progress. Most of the lindane-degrading bacterial isolates were Gram-negative bacteria. Interestingly, an increase of Gram-negative bacteria has been reported in marine sediment subjected to various stress conditions, among which are polycyclic aromatic hydrocarbons PAH [61,62,63] and trace element contamination [64]. The resistance to stressors and survival of Gram-negative bacteria is mainly attributable to the presence of cyclopropyl fatty acids FAs that maintain higher membrane stability [65].

In particular, among the selected strains showing lindane-removal capability, the isolate Li-6 was related to the species *Alteromonas australica*. Previous studies have reported that the genus *Alteromonas* is one of the most common heterotrophic bacteria living in marine habitats [66,67]. The hydrolytic activities of the *Alteromonas* genus as a whole are impressive, and this example fit with its known capabilities to exploit sudden inputs of organic matter in their environment. Indeed, members of the genus *Alteromonas* can grow rapidly as soon as a new carbon source is added to the marine habitat. On account of this feature, a broad diversity of carbon-processing pathways are ascribable to members of the genus *Alteromonas* including polycyclic aromatic hydrocarbon (PAH) biodegradation in crude oil-contaminated sediments [68,69]. Consistent with this high metabolic capability, we demonstrated the ability of *A. australica* to degrade lindane. Interestingly, the *Alteromonas* sp. belongs to the bacteria also associated with the marine sponge *Fasciospongia cavernosa*, and showed heavy metal resistance patterns [70].

The isolate Li-7 was assigned by 16S rRNA gene sequencing to the species *Mameliella phaeodactyli*, isolated for the first time from the marine algae *Phaeodactylum tricornutum* in 2013 [50]. This species, as well as Li-13 which is closely related to *Sulfitobacter dubius*, belongs to the family of Rhodobacteraceae in the class Alphaproteobacteria [71,72]. Interestingly, several Rhodobacteraceae members and the relative presence of members of *Sulfitobacter* have previously been identified in oil-polluted marine environments and have been shown to dominate oil-polluted environments of the North Sea [73] and Southeast Asia [74] and cold habitats [75,76]. In addition, members of Rhodobacteraceae have very diverse metabolisms and are also known to degrade hydrocarbons [77] and to possess haloalkane dehalogenases (HLDs) that catalyse the conversion of halogenated alkanes into their corresponding alcohol products and hydrogen halides. This suggests potential applications in biocatalysis, biosensors and cell imaging, as well as in the bioremediation of recalcitrant and carcinogenic halogenated by-products from organic synthetic reactions and halogenated pesticides and insecticides [78,79]. Moreover, representatives of *Sulfitobacter* can be considered as marine bacteria playing an important role in organic sulfur cycling [80].

The isolate Li-8 was assigned to the species *Pseudovibrio ascidiaceicola*. The association between sponges and *Pseudovibrio* spp.-related bacteria has been investigated in several studies. The first indications of the symbiotic character of this genus were published by Webster and Hill [41], who found a *Pseudovibrio* sp. as the dominating culturable alpha-proteobacterium associated with the sponge *Rhopaloeides odorabile*. The strain was localized in the mesohyl region of the sponge surrounding the choanocyte chambers. Indicative of a symbiotic life style, the *Pseudovibrio* strain was neither found in water sampled adjacent to the sponge, nor in diseased sponges sampled in the same region. The hypothesis of Webster and Hill [41] was supported by Enticknap et al. [39], showing the presence of a *Pseudovibrio* sp. in larvae of the sponge *Mycale laxissima*. Up to now, wide diversity of *Pseudovibrio* sp. have been isolated from sponges in Ireland [81], the Mediterranean Sea [82] and Brazil [83]. However, the physiology of the genus *Pseudovibrio* is insufficiently studied and hardly anything is known about its relevance in the environment and its role in the symbiosis with sponges. The present study represents a contribution to this topic, since we demonstrated the capability of *P. ascidiaceicola* to degrade lindane. This observation is in accordance with recent genome analyses of two *Pseudovibrio* sp. strains revealing their high metabolic versatility and their ability to use a wide range of organic substrates [84]. Interestingly, *Pseudovibrio* strains represented 54% of all the bacteria (64% of the Proteobacteria) isolated from the sponge *S. officinalis* on metal-enriched microbiological media, and were tolerant to at least one of the tested metals. These multiple heavy-metal resistant bacteria could survive well in a heavy metal-contaminated environment and thus can be also exploited for the remediation of metals from such an environment.

The isolate Li-12 was assigned to the species *Oceanicaulis stylophorae*. The genus *Oceanicaulis*, first proposed by Strömpl et al. [85], belongs to the family Hyphomonadaceae of the order Rhodobacterales (Alphaproteobacteria) [86]. At present, the genus *Oceanicaulis* comprises only two species with a validly published name, *Oceanicaulis alexandrii,* isolated from a non-toxigenic culture of the dinoflagellate *Alexandrium tamarense* [85], and *Oceanicaulis stylophorae*, isolated from the reef-building coral *Stylophora pistillata*, collected from seawater off the coast of southern Taiwan [52]. Members of the genus *Oceanicaulis* are characterized as Gram-negative, rod-shaped or vibrioid, aerobic, chemoheterotrophic and dimorphic, either non-motile with stalks (or prosthecae), or non-stalked and motile by means of a single polar flagellum. Up to now, our findings represent the first record of the genus *Oceanicaulis* from sponges.

The isolate Li-17 was strictly related to the species *Bacillus aquimaris*. *Bacillus aquimaris* exerts a broad metabolic versatility. A strain of this bacterial species, which was isolated from the soft coral *Sinularia* sp., was indeed found to degrade granular starch of various sources with the production of small oligosaccharides up to maltohexaose [87]. Moreover, recent studies have shown that *B. aquimaris*, with its organic tolerance to solvents such as acetone, methanol and benzene and its virtue of being a source of highly stable alkaline cellulase, can play a key role in industrial processes involving biphasic organic-aqueous fermentation and bioremediation of hydrocarbon-saturated environments [88].

Finally, the isolate Li-18 was assigned to the species *Ruegeria atlantica*. Biodegradation studies [89] evinced the capability of this species to effectively utilize spent engine (SE) oil as a carbon source. The FTIR spectra and GC–MS analysis confirmed the breakdown of aromatic and aliphatic hydrocarbons, and accumulation of degraded metabolites. The rapid utilization and degradation of complex hydrocarbons emphasizes the potential application for bioremediation process in marine environments.

Although sponge-microbial associations have long been documented and probably date back to Precambrian times about 500 million years ago [90], relatively little is known about the nature of the interactions. The bacterial strains isolated from *H. perlevis* able to degrade lindane presumably establish a symbiotic relationship with the sponge, suggesting the fascinating hypothesis that they constitute a consortium of symbiotic bacteria which could act simultaneously and probably synergistically in the degradation of the pesticide inside the sponge. Up to now, experiments were performed with a consortium of 10 bacterial species able to degrade HCH isomers, comprising one species of each of the genera *Flavobacterium*, *Vibrio* and *Burkholderia* and seven species of *Pseudomonas*. This consortium degraded nearly 90% of γ-HCH within 72 h of incubation [91]. However, the long-term impact of introducing microorganisms into the environment represents one of the issues related to this process that should be considered. This problem is overcome in the case of *H. perlevis*, since the sponge itself represents a microcosm hosting symbiotic lindane-degrading bacteria and survives in polluted environments. Therefore, the use of this natural microbial community associated with the sponge to clean a polluted marine environment can be viewed as “green technology” because it does not introduce exogenous bacteria into the waters to be restored. Obviously, the present paper represents a preliminary step toward the practical application in the field of this “green technology,” based on the development of a *H. perlevis* spongiculture in sites subjected to lindane pollution. Further studies are needed to increase knowledge of the sponge microbial consortium dynamic, activity and efficiency both in the laboratory in presence of other carbon sources and in lindane-polluted sites. Considering this, it is noteworthy that a sponge culture system of *H. perlevis* integrated in a mussel farm was already realized [92]. In particular, sponge fragments were obtained from adult sponge specimens coming from a wild population and transplanted within the mussel farm, placed on vertical rearing structures attached to a circular floating structure. Sponge cuttings and rearing techniques were made according to Corriero et al. [93]. Thus, a field pilot experiment, in which the efficiency of bacteria associated with *H. perlevis* in removing lindane will be proved, could represent the next step towards the development of tools for the recovery of lindane pollution. This action and future investigations will be preparatory to encourage further applicative bioremediation practices on a large scale in order to promote lindane restoration. Last but not least, exploring this bacterial diversity as well as the metabolic capabilities of these symbiotic bacteria represents an interesting challenge showing a great potential for diverse applications including biotechnologies.

## 4. Materials and Methods

### 4.1. Bacterial Isolates

The analysis was carried out on seven bacterial isolates from the sponge *H. perlevis* subjected to lindane exposure (concentration of 1 μg/L) during previous studies [44]. Briefly, after 7 days, lindane-exposed specimens of *H. perlevis* were washed with AS (seawater filtered on 0.22 µm pore size), gently squeezed with a glass stick and then the sponge pieces were homogenized in a sterile Waring blender. Afterwards, 100 μL of undiluted homogenate and serial dilutions (10^−1^, 10^−2^, 10^−3^, 10^−4^) were plated in triplicate on Bacto Marine Agar 2216 (Difco) and incubated at 22 °C for 7 days. At the end of the incubation period all the colony types were isolated and sub-cultured on Bacto Marine Agar. In particular, the isolates previously designated as Li-6, Li-7, Li-8, Li-12, Li-13, Li-17 and Li-18 were utilized on account of their evidenced capability to remove lindane.

### 4.2. Screening of Lindane Degradation by Bacterial Isolates

In order to further characterize the lindane-degrading microorganisms arising from the sponge homogenates, we tested their ability to grow in the presence of lindane for 12 days. Briefly, a set of three tubes containing 5 mL of a lindane solution (0.05 mg/L) in AS were prepared for each bacterial strain. A 0.1 mL aliquot of the bacterial suspension previously incubated in Marine Broth was added to each tube of the set. *Sphingobium japonicum* MTCC 6362 was procured from Microbial Type Culture Collection (MTCC), Institute of Microbial Technology (IMTECH), Chandigarh, India, and this was used as a reference strain in the experiment as a positive control added (0.1 mL of bacterial suspension) to each of the three tubes containing the lindane solution. A further set of three tubes containing only the lindane solution in AS at the same concentration represented the negative control. The tubes were sealed and placed in a test tube roller. After 12 days at 22 °C, bacterial growth was qualitatively determined by recording the optical density of each test tube at 560 nm. The tubes were centrifuged at 5000 × rpm and the resulting supernatants were diluted 1:10 with AS before being subjected to SPME-GC-MS analysis to evaluate the residual lindane concentration.

### 4.3. SPME-GC-MS Analysis

The solid phase microextraction (SPME) device and 100 μm thick polydimethylsiloxane (PDMS) coated fibers were supplied by Supelco (Bellefonte, PA, USA).

The gas chromatography–mass spectrometry system (GC–MS) consisted of a Finnigan TRACE GC ultra gas chromatograph equipped with a split/splitless injector coupled to an ion trap mass spectrometer (MS) (Finnigan PolarisQ) (Thermo, San Jose, CA, USA). A Supelco SPB-5 fused silica capillary column (30 m × 0.25 μm i.d., 0.25 μm film thickness) was used, with helium as a carrier gas (flow rate 1 mL/min). Before use, each fiber was conditioned in the GC injector at 300 °C for 3 h, as suggested by the supplier. Samples (15 mL) were placed in 15 mL amber vials and the vials sealed with hole caps and Teflon-faced silicone septa (Supelco). Extraction was carried out at 50 °C for 30 min by the direct immersion of the fiber in the solution under magnetic stirring. Thermal desorption (5 min desorption time) was performed into the GC injection port at 275 °C. To eliminate carry over, the fiber was subjected to a second thermal desorption after each chromatographic run.

The oven temperature program was: 50 (5 min)–180 °C at 12 °C/min, 180–230 °C at 5 °C/min, 230–245 °C at 2 °C/min. The GC transfer line was maintained at 250 °C. The mass spectrometer was operated in the electron impact positive ion mode (EI+) with the ion source temperature at 250 °C. The electron energy was 70 eV and the filament current 150 μA. Mass spectra were acquired in the *m*/*z* range 50–300. The detection of lindane was also accomplished in selected ion monitoring (SIM) mode, using the *m*/*z* ions 181 and 183.

### 4.4. 16S rRNA Gene Sequence Analysis of Lindane-Degrading Bacterial Isolates

In order to extract the total high-molecular-weight genomic DNA from lindane-degrading bacteria isolated in pure cultures, each colony isolate was inoculated in Marine Broth at 28 °C under rotary shacking until logarithmic phase (about 30 h). After this incubation time, each bacterial culture was pelleted (3000 rpm, 20 min) and processed as described by Talà et al. [94]. Then, total nucleic acids were extracted by phenol:chloroform:isoamylic alcohol (25:24:1 (*v*:*v*:*v*)) method according to standard procedures [95]. Finally, 15 μg·mL^−1^ ribonuclease A was used to remove contaminant RNA and subsequently, high molecular-weight DNA was collected after ethanol precipitation at −20 °C overnight and its purity was checked on 1% (*m*:*v*) agarose gel stained with ethidium bromide [95].

### 4.5. 16S rRNA Gene Sequencing and Phylogenetic Analyses

To assign taxonomy to our bacterial isolates, extracted DNAs were subjected to polymerase chain reaction (PCR) to amplify their 16S rRNA-encoding genes by using the following three primer pairs: 16SE20-42-F/16SEB683-R (corresponding to *E. coli* positions 20 to 683) [96], Com1-F/Com2-R (corresponding to *E. coli* positions 519 to 926) [97], and 16SEB785-F/16SEB1488-R (corresponding to *E. coli* positions 785 to 1488) [96]. These primer pairs amplified concatenated (and partially overlapping) DNA regions. PCRs were performed as follows: initial denaturation at 94 °C for 3 min, followed by 35 cycles of denaturation at 94 °C for 1 min, annealing for 1 min at 55 °C and extension at 72 °C for 1–2 min, and the final elongation step at 72 °C for 5 min. They were carried out in a Perkin-Elmer Cetus DNAThermal Cycler 2400. PCR products were isolated through 1% (wt./*v*) agarose gels in 1X TAE buffer (40 mM Tris–acetate, 1 mM EDTA, pH 8.0), recovered using the Qiaex II Gel extraction kit (Qiagen, Hilden, Germany). PCR products finally were sequenced as a service by MWG Biotech Custom Sequencing Service (Ebersberg, Germany). All sequences were compared with those of closely related reference strains using the EzTaxon-e server [48]. Multiple sequence alignments were performed with CLUSTAL W [98] at the Kyoto University Bioinformatic Center (Kyoto, Japan) using the following default settings. The CLUSTAL W output file was used to construct evolutionary trees with the SeaView 4 program [99] in accordance with the Neighbor-Joining (NJ) method [100]. Evolutionary distances were calculated with the NJ method in accordance with the algorithm of Kimura’s two parameter model [101]. Tree robustness was assessed by bootstrap resampling (1000 replicates each) [102].

### 4.6. 16S rRNA GenBank Accession Number

The 16S rDNA nucleotide sequences of the seven lindane-degrading bacterial isolates were deposited at GenBank with the following accession numbers: *Alteromonas* sp. Li-6 (KY287238), *Mameliella* sp. Li-7 (KY287239), *Pseudovibrio* sp. Li-8 (KY287240), *Oceanicaulis* sp. Li-12 (KY287241), *Sulfitobacter* sp. Li-13 (KY287242), *Bacillus* sp. Li-17 (KY287243), and *Ruegeria* sp. Li-18 (KY287244).

## 5. Conclusions

In conclusion the present study on the bacteria associated with *H. perlevis* capable to degrade lindane represents a prelude to the development of future strategies for the in situ bioremediation of this pollutant.

## Figures and Tables

**Table 1:** The bacterial strains showing the best growth and lindane removal performances after 12 days of lindane exposure.

Bacterial Isolate	Closest Species	Similarity (%)	Lindane Residual (%)
			(12 Days)
Control			89 ± 4
Li-6	*Alteromonas australica *CIP 109921^T^	97.23	3 ± 0.4
Li-7	*Mameliella phaeodactyli *KCTC 42178^T^	99.63	11 ± 0.6
Li-8	*Pseudovibrio ascidiaceicola *NBRC 100514^T^	100	5 ± 0.5
Li-12	*Oceanicaulis stylophorae *LMG 2723^T^	99.34	10 ± 0.5
Li-13	*Sulfitobacter dubius *KMM 3584^T^	98.34	3 ± 0.3
Li-17	*Bacillus aquimaris *JCM 11544^T^	98.67	13 ± 0.7
Li-18	*Ruegeria atlantica *NBRC 15792^T^	99.12	12 ± 0.7

Data are the mean ± SD (*n* = 3).

## Supplementary Materials

The supplementary materials are available online at the link: www. mdpi.com/1660-3397/15/4/108/s1.
