# Supplementary figures and images for: Lindane Bioremediation Capability of Bacteria Associated with the Demosponge Hymeniacidon perlevis
Source: Mar Drugs. 2017 Apr 6;15(4):108. doi: 10.3390/md15040108 (PMC5408254; doi:10.3390/md15040108)

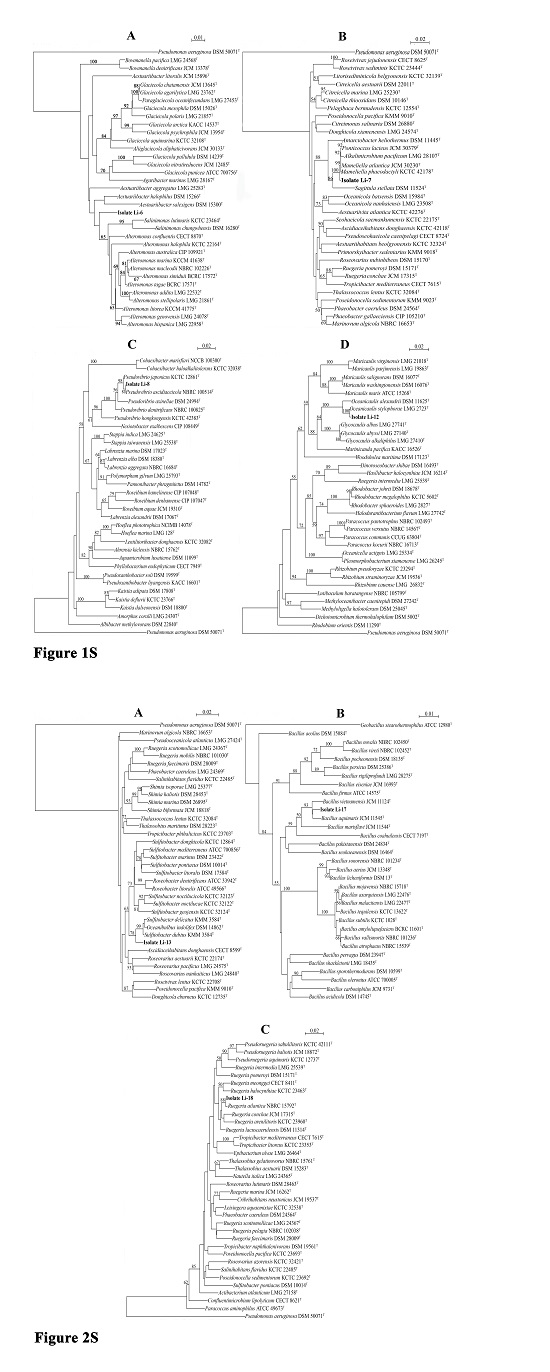

Supplement: Supplementary file 1 [file marinedrugs-15-00108-s001.jpg]
